# The AGE-RAGE Axis: Implications for Age-Associated Arterial Diseases

**DOI:** 10.3389/fgene.2017.00187

**Published:** 2017-12-05

**Authors:** Laura M. Senatus, Ann Marie Schmidt

**Affiliations:** Diabetes Research Program, Division of Endocrinology, Diabetes and Metabolism, Department of Medicine, New York University School of Medicine, New York, NY, United States

**Keywords:** RAGE, glycation, arterial diseases, inflammation, aging

## Abstract

The process of advanced glycation leads to the generation and accumulation of an heterogeneous class of molecules called advanced glycation endproducts, or AGEs. AGEs are produced to accelerated degrees in disorders such as diabetes, renal failure, inflammation, neurodegeneration, and in aging. Further, AGEs are present in foods and in tobacco products. Hence, through both endogenous production and exogenous consumption, AGEs perturb vascular homeostasis by a number of means; in the first case, AGEs can cause cross-linking of long-lived molecules in the basement membranes such as collagens, thereby leading to “vascular stiffening” and processes that lead to hyperpermeability and loss of structural integrity. Second, AGEs interaction with their major cell surface signal transduction receptor for AGE or RAGE sets off a cascade of events leading to modulation of gene expression and loss of vascular and tissue homeostasis, processes that contribute to cardiovascular disease. In addition, it has been shown that an enzyme, which plays key roles in the detoxification of pre-AGE species, glyoxalase 1 (GLO1), is reduced in aged and diabetic tissues. In the diabetic kidney devoid of *Ager* (gene encoding RAGE), higher levels of *Glo1* mRNA and GLO1 protein and activity were observed, suggesting that in conditions of high AGE accumulation, natural defenses may be mitigated, at least in part through RAGE. AGEs are a marker of arterial aging and may be detected by both biochemical means, as well as measurement of “skin autofluorescence.” In this review, we will detail the pathobiology of the AGE-RAGE axis and the consequences of its activation in the vasculature and conclude with potential avenues for therapeutic interruption of the AGE-RAGE ligand-RAGE pathways as means to forestall the deleterious consequences of AGE accumulation and signaling via RAGE.

## Introduction

Our population is aging and living longer. Although the average life expectancy of the population is increasing in the United States, there remain significant consequences of the aging process. An average 1 death every 40 s is due to cardiovascular disease (Mozaffarian et al., 2016). The prevalence of heart failure is highest among the adult population 65 years or older (Lakatta and Levy, 2003). As one in four, or 25% of individuals, will be over 65 years of age in the United States by the year 2035, these statistics underscore the fact that significant increases in heart failure are to be expected over this time frame (Lakatta and Sollott, 2002). Clinical and experimental evidence suggests that natural aging imbues inherent risk for the development of cardiovascular complications, such as arterial stiffness, atherosclerosis, and hypertension, which eventually may lead to myocardial infarction, stroke, and heart failure (Safar et al., 2003; Izzo, 2004; Sethi et al., 2014). Hence, understanding the underlying mechanisms may aid in the discovery of new therapeutic approaches to deter pathological vascular aging.

Changes in the components of large arteries due to advancing age have been described in humans and animals (Spinetti et al., 2004; Pepe and Lakatta, 2005). Age-associated blood vessel remodeling includes such features as dilation of the lumina, intimal and medial thickening, changes in the extracellular matrix (ECM), and augmented stiffness (Gaballa et al., 1998). In addition to these structural changes, other mechanisms contribute to the overall consequences of aging to the arterial wall, including such phenomena as inflammation, endothelial dysfunction, and oxidative stress (Xu et al., 2003). Fibroblasts and smooth muscle cells (SMC) contribute to aging in the vasculature, in part by increasing ECM; macrophages contribute by increasing inflammatory factors that have a wide range of possible consequences, such as vascular hyperpermeability and an increase in the procoagulant state (Sprague and Khalil, 2009; Strait and Lakatta, 2012). These pathobiological events adversely affect the vessel wall and all of its components (Najjar et al., 2005; Greenwald, 2007), potentially contributing to arterial aging.

It has been shown that the aged human arterial wall exhibits a more proinflammatory signature, with increased expression and activity of matrix metalloproteinases (MMPs) and chemokines (Wang et al., 2007). Atop these considerations is the effect of co-morbid conditions in aging, which may augment production of inflammatory mediators and exacerbate the impact of arterial aging, examples of which include diabetes mellitus (types 1 or 2 or the rarer forms of diabetes); chronic renal disease; and chronic immune/inflammatory disorders.

## AGEs: production and functions in arterial aging

Advanced glycation endproducts (AGEs) are a diverse group of macromolecules and at least 20 different specific AGEs have been described to date. Among the major groups of AGEs are carboxymethyl lysine (CML), carboxyethyl lysine (CEL), pentosidine, glucosepane, methylglyoxal lysine dimer, glyoxal lysine dimer, and glycolic acid lysine amide (Henning and Glomb, 2016). AGEs form throughout life via the process of non-enzymatic glycation of proteins and lipids, and this process is accelerated during hyperglycemia, oxidative stress, aging, advanced renal disease, and inflammation (Daffu et al., 2013; Singh et al., 2014; Baig et al., 2017). Humans and animals are also exposed to exogenous sources of AGEs ingested through food-derived AGEs and tobacco products (Luevano-Contreras and Chapman-Novakofski, 2010; Uribarri et al., 2015). It has been shown that restriction in dietary AGE intake may increase the lifespan in animals (Cai et al., 2002; Luevano-Contreras and Chapman-Novakofski, 2010).

AGEs accumulate in aging tissues and on vulnerable plasma proteins. Higher levels of circulating AGEs have been linked to chronic diseases in aging subjects (Semba et al., 2015). The accumulation of AGEs is increased and accelerated in hypertensive subjects (McNulty et al., 2007) and is also associated with diabetes (Soulis et al., 1997; Yan et al., 2003). In fact, aged subjects, even though healthy, may have higher AGE accumulation compared to younger subjects with diabetes and its complications, thus underscoring that AGE production and accumulation accompanies the normal aging process (Hadi and Suwaidi, 2007). Therefore, multiple factors such as the rate of accumulation of AGE ligand, the absolute concentration of the ligand, and individual susceptibility to AGE formation may be important in determining an individual's AGE burden.

AGEs modify collagen and elastin in the vascular wall (Meerwaldt et al., 2004); because of reduced turnover of such proteins, they become more susceptible to glycation during the aging process (Schleicher et al., 1997; Sell and Monnier, 2012). Elevated levels of plasma CML-AGEs are associated with diastolic dysfunction in aging (Campbell et al., 2012). In experiments in diabetic rats, higher AGE crosslinking of collagen was associated with increased stiffness of the aorta (Reddy, 2004). This may change the beneficial functions of several important molecules of the ECM, which can mediate vascular dysfunction. Numerous studies have confirmed the correlation between AGE accumulation and increased artery stiffness (Goldin et al., 2006; Campbell et al., 2011). Arterial stiffness is associated with greater risk for aging-associated cardio- and cerebrovascular diseases and mortality (Laurent et al., 2001; Mattace-Raso et al., 2006; Kaess et al., 2012). AGE accumulation causes upregulation of inflammation and destruction of collagen and elastin, along with other proteins of the ECM (Sims et al., 1996; Greenwald, 2007; Peppa and Raptis, 2008; Baulmann et al., 2010).

## Measuring AGEs

AGEs can affect virtually every tissue in the body, either through mediation of cellular damage via protein cross-linking and/or through their binding to cell surface receptors. Since various diseases have been linked to AGEs, it is plausible that AGEs can be utilized as biomarkers, such as for predilection to disease, state of disease activity, and/or response to therapeutic interventions.

Measurment of skin autofluorescence (SAF) estimates the skin tissue AGE content and may predict cardiovascular complications, at least in certain subjects (Lutgers et al., 2009; Noordzij et al., 2012; Tanaka et al., 2012). Some AGEs are fluorescent and can be non-invasively measured in skin by autofluorescence, as a representative marker of the total AGE modifications of other long-lived tissues in association with vascular disease. In a recent study, SAF was measured using an autofluorescent spectrometer followed by measurement of endothelial function and arterial stiffness. The results indicated that SAF was associated with increased arterial stiffness in the older individuals and that arterial function was blunted by the advancing age of the subject (Sturmer et al., 2015). It is important to note, however, that there are limitations to the use of SAF; first, it only measures fluorescence and not all AGEs are fluorescent; second, skin fluorophores exist that are not related to AGEs, and therefore, such measurement is not reflective of the AGE pool; third, in subjects with darker skin pigmentation, SAF measurements have been found to be less reliable, thereby possibly reducing the applicability of this technique across diverse groups of subjects; and fourth, certain skin creams, such as agents used to “tan” or “brown” the skin may cause direct interference with SAF measurements. Hence, although the technique does not require biopsies or invasive approaches, there are notable limitations that must be considered in its use (Da Moura Semedo et al., 2017).

Traditionally, the precise detection of AGE measurement includes HPLC (High-Performance liquid chromatography) (Ashraf et al., 2015) with fluorescence-based detection. Using HPLC methods, high levels of serum AGE, such as CML-AGE and pentosidine were shown to increase with age and in patients with diabetes (Aso et al., 2000). The use of ELISA (Enzyme-linked immunosorbent assay), an immunological technique for the determination of AGEs, proved to be an alternative for detection of AGEs in samples such as serum and plasma (Munch et al., 1997; Takeuchi et al., 1999). Tissue AGE concentrations using immunohistochemical methods can also be measured using antibodies to detect CML-AGE (Soulis et al., 1997). Using these methods, AGE have been identified in arterial disease and have been localized to early atherosclerotic plaques and cellular constituents, including macrophages and SMCs (Stitt et al., 1997). LC-MS (liquid chromatography- mass spectroscopy) is another technique for the detection of AGEs and the early glycation products. LC-MS allows for AGEs such as pyrraline to be detected in human skin and plasma in very low concentrations (picomolar range) (Pitt, 2009). The possibility of using AGE measurements to gauge the state of AGE-related disease activity and the effectiveness of therapeutic intervention underscores the importance of using reliable methods for the detection of AGEs.

## AGEs and their pathobiological actions: interactions with cellular receptor RAGE

RAGE (Receptor for Advanced glycation end products) was identified in 1992 from bovine lung as a protein that bound AGEs in a dose-dependent manner (Wautier et al., 1994). RAGE has many ligands that increase in aging, even beyond AGEs, and it is a cell surface macromolecule. RAGE contains extracellular domains composed of one V (variable)-type domain and two C (constant)-type immunoglobulin—like domains (C1 And C2); these are followed by a single transmembrane spanning helix (Koch et al., 2010), and the cytoplasmic domain, which is essential for signal transduction (Xue et al., 2014). RAGE binds a diverse group of ligands, including AGEs, at least certain members of the S100/calgranulins, high mobility group Box-1 (HMGB1), Mac-1, and amyloid- β peptide, particularly its oligomeric forms (Herold et al., 2007).

Mechanisms by which AGEs could alter the vasculature and increase arterial stiffness include generation of inflammation (Chavakis et al., 2004) and oxidative stress (Tan et al., 2007). Further, AGEs binding to endothelial cell surface RAGE can lead to stimulation of NADPH oxidase, thereby increasing the production of reactive oxygen species (ROS) (Wautier et al., 2001). Additional mechanisms such as mitochondrial stress may further increase the production of ROS (Rubattu et al., 2013; Li et al., 2014; Montezano and Touyz, 2014). Previous studies proposed that one of the mechanisms by which AGE/RAGE contributes to endothelial dysfunction is through regulation of the production and expression of tumor necrosis factor (TNF)-α. The transcription factor nuclear factor-κB (NF-κB), triggered by inflammation and by ROS, plays a key role in cytokine and inflammatory mediator expression, thereby exacerbating microvasculopathy and mediating pathological changes in gene expression, at least in part through RAGE ligand-RAGE interactions and activation of cellular signal transduction (Gao et al., 2008; Kay et al., 2016).

Evidence of RAGE-mediated perturbation *in vivo* has also been demonstrated. Diabetic *apolipoprotein E* (*ApoE*) deficient mice that are also devoid of *Ager* (gene encoding RAGE) display reduced atherosclerosis and lower expression of vascular cell adhesion molecule (VCAM)-1 and tissue factor (Kislinger et al., 2001). AGEs also induce vascular endothelial growth factor (VEGF) expression in microvascular endothelial cells (Yamagishi et al., 1997), which may have implications for the diabetic retina, as an example. In addition to the chronic conditions of AGE formation discussed above, such as aging, diabetes, and chronic inflammatory conditions, research has illustrated that AGEs may form rapidly in settings of acute stress as well. For example, endothelial cells subjected to *in vitro*-applied hypoxia release AGEs within minutes of exposure to reduced levels of oxygen (Chang et al., 2008). These considerations indicate that it was important to identify means to block AGE-RAGE interactions in the vasculature.

In animal studies, treatment with soluble RAGE (sRAGE), the soluble extracellular domains of RAGE, which sequester AGEs and RAGE ligands, thereby blocking their interaction with RAGE demonstrated significant reductions in atherosclerotic lesion area (Park et al., 1998), in a manner independent of changes in lipid or glucose levels. In other studies, sRAGE treatment in rodents significantly mitigated diabetic vascular hyperpermeability (Schmidt et al., 1999).

Hallam and colleagues demonstrated that aged 24 month-old Fischer 344 rats displayed higher vascular RAGE expression in the aorta, and higher expression of the polyol pathway enzyme, aldose reductase (AR), which stimulates metabolic pathways that increase AGE formation. Aging-related vascular dysfunction was evident in these rats on account of impaired endothelial relaxation in response to acetylcholine (Hallam et al., 2010). Treatment of aged Fischer 344 rats with sRAGE improved endothelial dependent relaxation in response to acetylcholine (Hallam et al., 2010). Taken together, these studies illustrate that increased AGE burden and RAGE expression mediate vascular dysfunction and that these perturbations may be suppressed by administration of antagonists of ligand-RAGE interactions *in vivo*.

## Glyoxalase 1 (GLO1) and amplification of AGE accumulation

In addition to increased direct mediators of damage in aging, defense mechanisms may also be attenuated in aging. (GLO1) contributes to the regulation of the levels of the pre-AGE methylglyoxal (MG) and MG-derived AGEs (Giacco et al., 2014). MG, formed mainly inside cells, is a potent glycating agent (Rabbani and Thornalley, 2014).

Published work has suggested a link between RAGE and *Glo1*. Exposure of cultured endothelial cells to high glucose increases expression of RAGE and various RAGE ligands, such as S100B, AGEs, and HMGB1; this was prevented by overexpression of *Glo1* (Brouwers et al., 2011, 2014). Reiniger and colleagues showed that renal accumulation of AGEs promotes kidney dysfunction and that when *Ager* is deleted in OVE26 diabetic mice, reduced pathological, and functional derangements in the kidney ensued, in parallel with reduced MG levels and higher levels of GLO1 in the kidney (Reiniger et al., 2010). These authors showed that in *Ager* null diabetic OVE26 kidney, levels of MG were lower than those of wild-type diabetic OVE26 controls, despite equal levels of high glucose. Reiniger and colleagues traced the mechanism to RAGE-dependent downregulation of *Glo1* mRNA and activity in diabetes (Reiniger et al., 2010). Thus, RAGE activation may perpetuate AGE accumulation and deletion of *Ager* may exert its protection, at least in part by downregulation of *Glo1*.

It is possible that increasing GLO1 expression and/or activity may slow down age-related damage, as acceleration in glycation in aged rats was attenuated by transgenic (Tg) expression of *Glo1* in these animals (Jo-Watanabe et al., 2014). Interestingly, exercise training in aged rats resulted in activation of GLO1, with consequent reduction in the formation of MG and CML, along with lower RAGE expression in the aorta (Gu et al., 2014). Overall, agents that augment GLO1 to block formation of AGEs may serve as therapeutic strategies for averting complications in vascular disorders in which AGEs accumulate.

## RAGE/DIAPH1 signal transduction axis: link to vascular dysfunction

RAGE requires its cytoplasmic domain for signal transduction. Hudson and colleagues demonstrated the interaction of the cytoplasmic domain of RAGE tail with mammalian diaphanous 1 or DIAPH1 (Hudson et al., 2008). The cytoplasmic domain or tail of RAGE (ctRAGE) binds specifically to the formin homology 1 (FH1) domain of DIAPH1 (Hudson et al., 2008). Formins are actin-binding molecules that contribute to Rho GTPase down-stream signals (Hudson et al., 2008) in cells such as vascular cells, monocytes/macrophages, and transformed cells. DIAPH1 has also been shown to be an effector of serum response factors (SRFs), which are linked to gene regulation mechanisms, and cellular signaling mechanisms such as AKT and GSK-3beta (Toure et al., 2012).

In SMCs, DIAPH1 was required for RAGE ligand (S100B)-induced c-Src translocation to the plasma membrane, RAC1 activation, generation of ROS and cellular migration. RAC1 modulates the actin cytoskeleton, the arrangement of which governs cell motility and regulates signal transduction pathways (Toure et al., 2012). To verify the RAGE-DIAPH1 interaction, Shekhtman and colleagues used NMR spectroscopy to identify the four key amino acids in the RAGE cytoplasmic domain (Q3, R4, R5, and Q6 corresponding to Q364, R365, R366, and Q367 of the full-length RAGE) that are essential for the interaction of ctRAGE with the FH1 domain of DIAPH1. When R5/Q6 were mutated to alanine residues and expressed in murine SMCs, AKT signaling and cellular migration and proliferation in response to RAGE ligand S100B, but not to non-RAGE ligands, were significantly reduced (Rai et al., 2012).

A role for DIAPH1 in RAGE signaling in macrophages has also been demonstrated. In macrophages devoid of *Diaph1*, hypoxia-mediated upregulation of the transcription factor *Egr1*, which upregulates inflammatory and prothrombotic mediators, was prevented (Xu et al., 2010). To test these points *in vivo* and the role of DIAPH1 in mediating the effects of RAGE ligands, studies are underway in animals of diabetes, aging, and vascular perturbation to probe the potential impact of DIAPH1 in vascular dysfunction.

**Figure 1:**
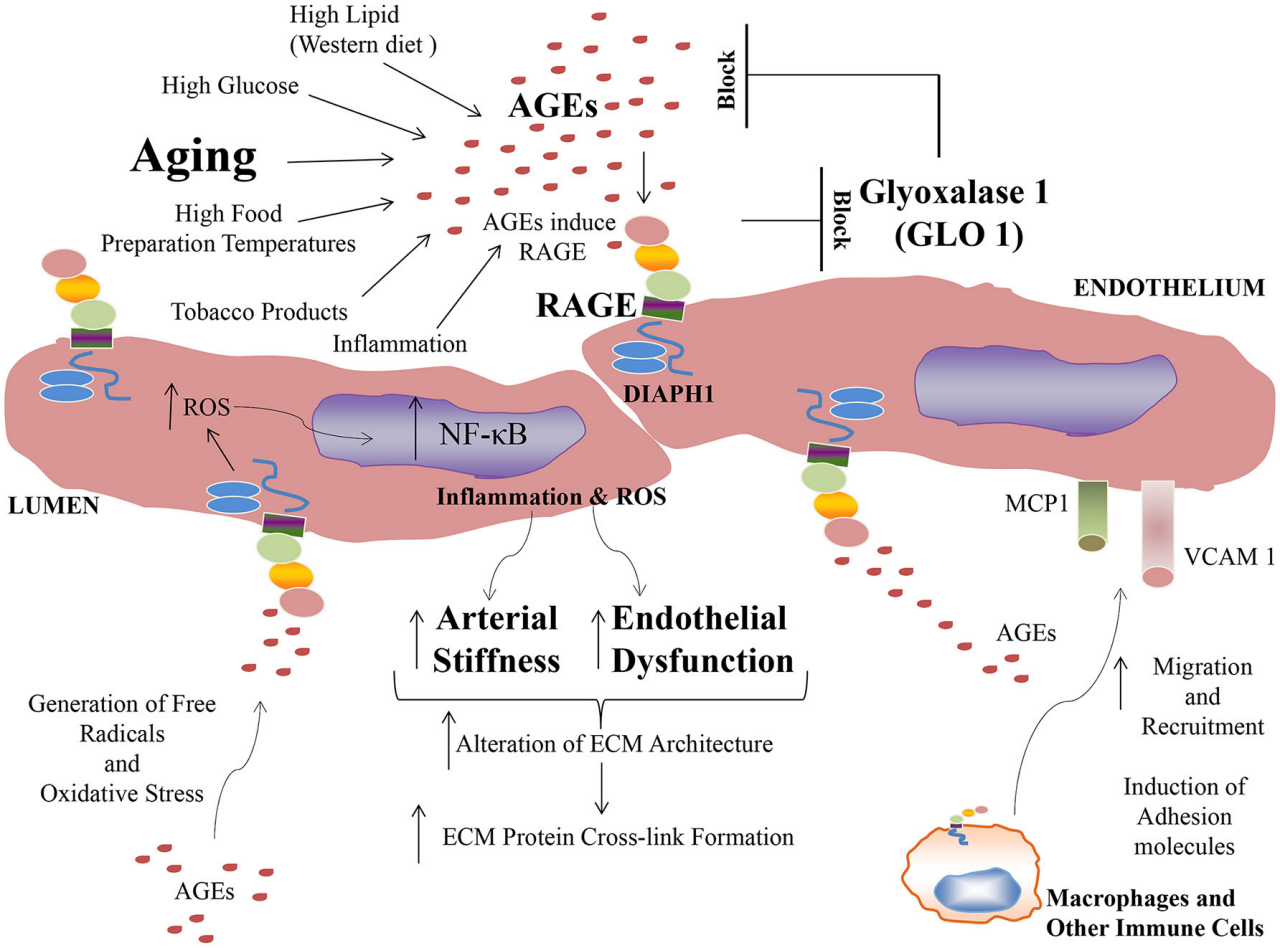
Formation of AGEs and mechanisms of their accumulation and pathobiological actions via the signal transduction receptor RAGE.

Taken together, extensive evidence is building to implicate AGEs and RAGE in the pathogenesis of vascular perturbation, which stimulate processes that lead to the development of arterial stiffness, an established harbinger of cardiovascular disease and aging. AGEs, via RAGE stimulate endothelial cells to generate ROS and to activate cellular signaling pathways, at least in part through the cytoplasmic domain of RAGE binding to DIAPH1; processes which lead to activation of seminal transcription factors such as NF-κB (Figure 1). In addition to AGE-RAGE activation of endothelial cells and mediation of endothelial dysfunction, AGEs, via RAGE, may also stimulate macrophages and other immune cells, to induce migration and recruitment of inflammatory cells into AGE-laden foci in the tissues. Further, research has shown that a natural anti-AGE mechanism, the enzyme GLO1, which detoxifies pre-AGE species, is downregulated by the actions of RAGE, such as in the diabetic kidney. Hence, AGE-RAGE activation stimulates a feed forward loop, in which AGE-RAGE interaction causes vascular perturbation and, in parallel, a mechanism to perpetuate AGE production and accumulation.

In the section to follow, we consider therapeutic opportunities in halting the detrimental actions of this AGE-RAGE pathway.

## Therapeutic strategies: targeting AGE and RAGE

**Figure 2:**
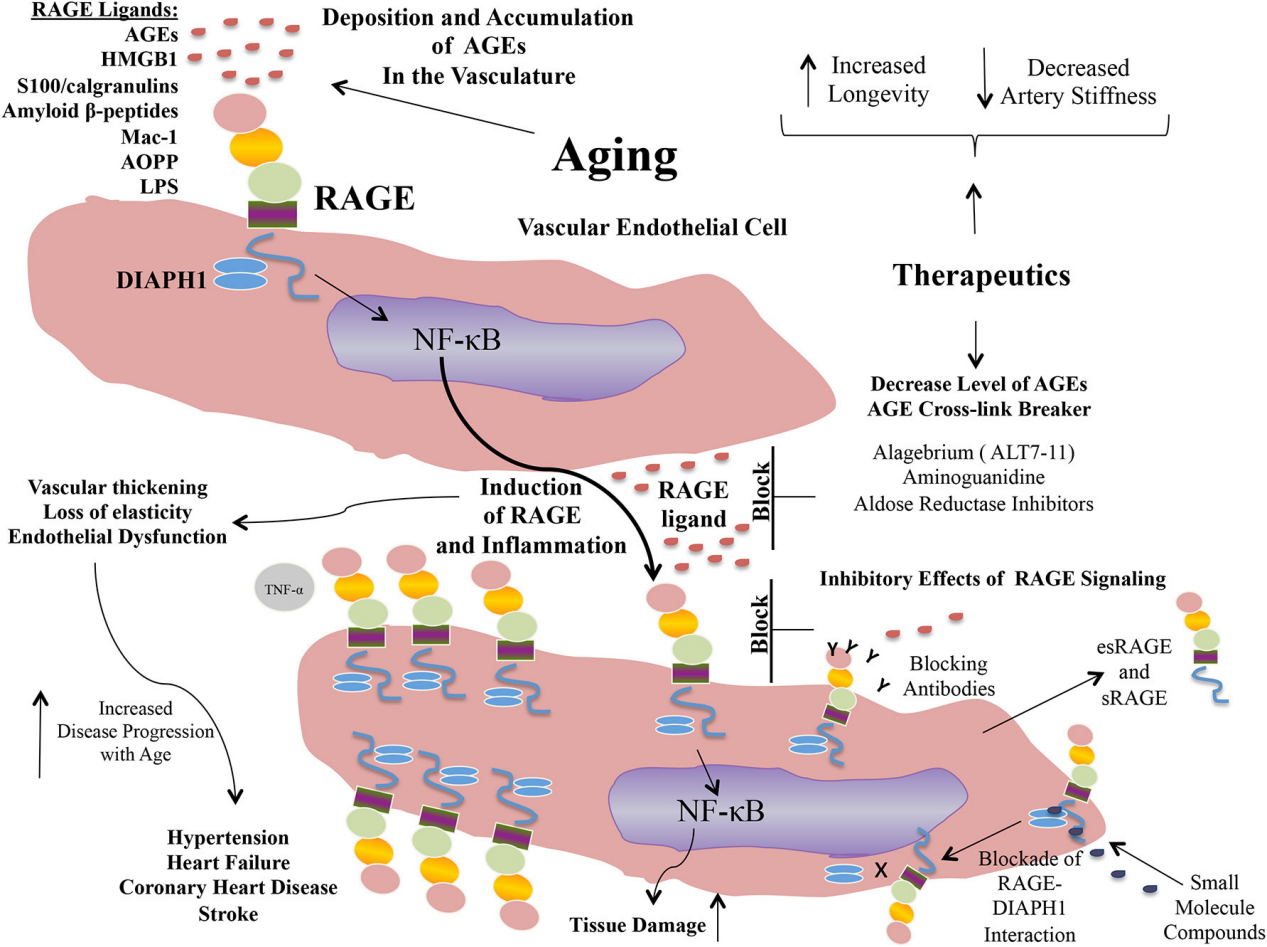
Targeting AGEs and RAGE: examples of putative therapeutic strategies.

Propelled by the epidemiological and experimental evidence linking AGE and RAGE to the pathogenesis of arterial stiffness and vascular perturbation, AGEs and RAGE have been identified as targets for therapeutic intervention in these settings. In the sections to follow, we detail examples of some of the strategies to block AGEs and RAGEs for their possible benefits in cardiovascular diseases (Figure 2).

### Anti-AGE strategies

Alagebrium, or ALT7-11, is an AGE cross-link breaker. In animals and humans, this agent improved ventricular function and arterial compliance (Kass et al., 2001; Vaitkevicius et al., 2001); reduced expression of RAGE and collagen accumulation in vascular tissues; and in patients with systolic hypertension, it improved endothelial function (Zieman et al., 2007). Although Alagebrium is no longer available, its use served as an important test of the AGE hypothesis in vascular stiffness and function.

Other anti-AGE strategies, such as aminoguanidine, which blocks AGE production, also had beneficial effects in increasing vascular elasticity and in augmenting left ventricular arterial coupling, as well as decreasing vascular permeability in diabetic rats (Wu et al., 2008). Atherosclerosis was attenuated in streptozotocin-induced diabetic *Apoe*-deficient mice treated with aminoguanidine (Forbes et al., 2004). Aminoguanidine also reduced AGE accumulation from food sources (He et al., 1999). Finally, other strategies to reduce AGEs are being investigated, such as aldose reductase inhibitors (ARI). ARI have been shown to suppress AGE accumulation in the atherosclerotic plaques and, in parallel, to reduce atherosclerotic plaque lesions (Vikramadithyan et al., 2005).

In summary, it is noteworthy that despite testing of multiple classes of anti-AGE agents, none have obtained, at least to date, approval for anti-AGE indications. Although there are many possible reasons for this, we propose that one reason is that solely targeting AGEs fails to capture the pathobiological effects of distinct RAGE ligands. Therefore, it is not surprising that attempts are underway to directly target RAGE as a therapeutic strategy.

### Anti-RAGE strategies

Approaches to limit RAGE ligand AGEs have been accompanied by efforts to block RAGE itself and these have been tested *in vitro* and *in vivo*; in addition, human clinical trial testing is also underway. *In vitro*, pre-treatment of AGE-stimulated endothelial cells with anti-RAGE antibodies or anti-oxidants blocked cellular perturbation (Yan et al., 1994). Another RAGE blocking agent currently in Phase III clinical trials in Alzheimer's disease is the small molecule Azeliragon, which inhibits the receptor for advanced glycation endproducts through its first extracellular V-type domain, which prevents RAGE ligands from interacting with RAGE. It is orally bioavailable (Sabbagh et al., 2011).

The *AGER* gene may be alternatively spliced to result in the generation of several RAGE isoforms (Yonekura et al., 2003; Kalea et al., 2009). The C- terminally truncated RAGE (known as endogenous secretory RAGE (esRAGE) or RAGEv1, does not contain the transmembrane domain, and is secreted. Other forms of soluble RAGE also exist, as the cell surface RAGE can be proteolytically cleaved by MMPs or ADAM10, thereby resulting in the release of soluble RAGE (sRAGE) (Hanford et al., 2004).

In mouse models, sRAGE treatment suppressed acceleration and blocked the progression of established atherosclerosis in diabetic *Apoe* null mice (Park et al., 1998; Bucciarelli et al., 2002). Various studies in human subjects have sought to determine whether the plasma sRAGE or esRAGE level is associated with cardiometabolic diseases (Choi et al., 2009). Generally, plasma sRAGE/esRAGE levels are lower in subjects with these disorders vs. healthy controls. Thus, recombinant sRAGE might be of benefit in arterial aging and metabolic diseases.

Beyond targeting RAGE and the extracellular domains, distinct therapeutic opportunities have arisen regarding RAGE signaling via blockade of RAGE-DIAPH1 interaction. Manigrasso and colleagues developed a high throughput RAGE tail-DIAPH1 binding assay and screened a library of >58,000 small molecule compounds to identify molecules that blocked this interaction. A series of 13 compounds was identified that exhibited high affinity binding to ctRAGE domain. *In vitro* and *in vivo* studies illustrated that these compounds displayed inhibitory effects on RAGE signal transduction in SMCs *in vitro*, and *in vivo*, on RAGE ligand-stimulated inflammatory gene expression in liver and kidney tissue (Manigrasso et al., 2016). Therefore, the discovery that the cytoplasmic domain of RAGE bound DIAPH1 may aid in the identification of a distinct class of RAGE signaling intracellular antagonists.

## Conclusions and perspectives

Evidence is accruing that exposure to AGEs contributes to detrimental aging-related outcomes and to reduced health and life span. *In vitro* and *in vivo* animal model studies have shown that AGEs affect and disrupt cellular and tissue homeostasis. AGEs can cause alteration of ECM architecture, thereby affecting cellular permeability and signaling; mediate ECM and circulating protein cross-linking; and they can activate cellular signaling and modulate transcription factor activities and subsequent gene expression via receptors such as RAGE. AGE accumulation may result in the increased expression of RAGE in a ligand-enriched environment and exacerbate proinflammatory mechanisms, thereby accelerating aging-associated arterial diseases.

RAGE is expressed on a number of important cell types implicated in arterial aging and vascular pathology. Once AGEs are formed, albeit by diverse intrinsic and environmentally-triggered mechanisms, their interaction with RAGE on endothelial cells, SMCs, and immune cells such as macrophages, results in upregulation of inflammatory and oxidative stress-provoking factors, thereby providing a mechanism to link AGE-RAGE to arterial aging and its consequences, such as stroke, hypertension, atherosclerosis, myocardial infarction, and heart failure. Of note, as hyperglycemia accelerates AGE formation, these AGE-RAGE processes are amplified in diabetes. Epidemiological studies assuredly support the exacerbation of cardiovascular disease in subjects with diabetes.

Certainly, more research is required to understand the entire scope of RAGE signaling and the extent to which blocking AGEs/RAGE/DIAPH1 interaction may intercept the full pathobiology of RAGE activation. Key remaining questions include whether interventions to reduce AGEs and/or to block RAGE extracellular and/or intracellular domains, might provide the greatest protection in attenuation of arterial aging and vascular dysfunction (See Table 1 for a summary of some of the key anti-AGE and anti-RAGE therapeutic approaches). Clinical studies to address these concepts are required to optimize strategies to protect the vasculature from the adverse effects of AGEing.

**Table 1:** Key anti-AGE and anti-RAGE therapeutic approaches.

**Examples of anti-AGE and anti-RAGE therapeutic strategies**	**Biochemical target/Mechanisms of action**
Alagebrium (ALT7-11)	AGE cross-link breaker
Aldose reductase inhibitors (ARI)	Blockade of Aldose Reductase on glucose metabolism that contributes to AGE formation
Aminoguanidine	Blockade of AGE production
Azeliragon	Small molecule inhibitor of RAGE ligand binding to the RAGE extracellular V-domain
Anti-RAGE antibodies	Blockade of ligand binding to RAGE
Inhibitors of RAGE tail-DIAPH1 binding	Blockade of RAGE signal transduction
Upregulation/Activation of glyoxalase-1	Augmentation of detoxification of AGE precursors
Soluble RAGE (sRAGE) or Endogenous secretory RAGE (esRAGE)	RAGE ligand binding species that sequester RAGE ligands and block their biological effects
Vitamin C, Vitamin E	Anti-oxidants, possible effects on reduction of AGE formation and AGE effects

## Materials and methods

Search Strategies
Arterial Aging:
11,140 refs
https://www.ncbi.nlm.nih.gov/pubmed/?term=arterial+aging
Arterial aging and glycation:
116 refs:
https://www.ncbi.nlm.nih.gov/pubmed/?term=arterial+aging+and+glycation
Arterial aging and advanced glycation end product:
95 refs:
https://www.ncbi.nlm.nih.gov/pubmed/?term=arterial+aging+and+advanced+glycation+end+product
Arterial aging and receptor for advanced glycation end products
22 refs:
https://www.ncbi.nlm.nih.gov/pubmed/?term=arterial+aging+and+receptor+for+advanced+glycation+end+products.

## Author contributions

LS and AS: Wrote and edited the manuscript.

### Conflict of interest statement

The authors declare that the research was conducted in the absence of any commercial or financial relationships that could be construed as a potential conflict of interest.

## Abbreviations

AGEs: advanced glycation endproducts
ALT7-11: Alagebrium
AR: aldose reductase
ARI: aldose reductase inhibitors
CML: carboxymethyl lysine
CEL: carboxyethyl lysine
ctRAGE: cytoplasmic domain of RAGE
DIAPH1: diaphanous-1
ELISA: enzyme-linked immunosorbent assay
esRAGE: endogenous secretory RAGE
ECM: extracellular matrix
FH1: formin homology 1
Glo1: Glyoxalase 1
HMGB1: high mobility group Box-1
HPLC: high performance liquid chromatography
LC-MS: liquid chromatography tandem mass spectrometry
MG: methylglyoxal
ROS: reactive oxygen species
VCAM-1: vascular cell adhesion molecule−1
VEGF: vascular endothelial growth factor
RAGE: receptor for advanced glycation end products
SAF: Skin autofluorescence
SMC: smooth muscle cells
sRAGE: soluble RAGE
SRFs: serum response factors
Tg: transgenic.
